# An Atypical Presentation of Cystic Echinococcosis

**DOI:** 10.5811/cpcem.2020.1.45842

**Published:** 2020-02-24

**Authors:** Bhavana Tetali, Daniel C. Grahf, Elian D. Abou Asala, Daniel Axelson

**Affiliations:** * Henry Ford Health System, Department of Internal Medicine, Detroit, Michigan; † Wayne State University School of Medicine, Detroit, Michigan; ‡ Henry Ford Health System, Department of Emergency Medicine, Detroit Michigan

## Abstract

Cystic echinococcosis (CE) is an infection caused by the *Echinococcus granulosus* tapeworm. CE generally manifests in the liver, but it may present in any organ. These patients often first present to the emergency department. Mortality over 10 years is significant for those who go undiagnosed. We report the case of a 34-year-old patient who immigrated from Yemen six years earlier. She presented with acute onset dysuria, suprapubic pain, and fever. Imaging revealed a primary multicystic mass on the right renal pole with a secondary lesion in the right hepatic lobe. On further investigation, the patient’s serum was positive for echinococcus antibodies.

## INTRODUCTION

Cystic echinococcosis (CE) is an infection caused by the *Echinococcus granulosus* tapeworm, which results in the creation of cystic structures within a range of visceral organs. Ninety percent of these cysts are in the liver or lungs. The cysts can be filled with thousands of “brood” capsules that in definitive hosts evaginate and invade surrounding host tissues. The definitive host is most often canines with intermediate hosts being sheep, cattle, and pigs.^1^ Humans are incidental hosts for CE; they most frequently ingest eggs via the fecal oral route from contaminated food or water. In the United States, cases are quite rare and most are found in immigrants from endemic countries. It is estimated that these cysts enlarge by about 1–5 centimeters (cm) per year, although rates are highly variable.^2^,^3^ Patients may remain asymptomatic for years. Those that develop symptoms may go undiagnosed for a long period of time, especially given the low prevalence of CE in the US and the nonspecific symptoms that arise. If left unidentified and untreated, mortality from CE is estimated to be 90% by 10 years.^4^

In this case, we discuss a patient who presented to the emergency department (ED) with genitourinary complaints and was diagnosed with cystic echinococcosis (CE) of primary renal involvement, a rare anatomic location. The case exemplifies the diagnostic workup and acute management of extrahepatic CE in the ED in a high-risk patient.

## CASE REPORT

A 34-year-old female with no past medical history presented to the ED complaining of dysuria, suprapubic pain, generalized myalgias, and subjective fever for one-day duration. The patient denied recent travel, sick contacts, or contact with animals, but she had immigrated from Yemen six years prior. On presentation, the patient was febrile to 38.8º Celsius and tachycardic to 133 beats per minute. Complete blood count and lactic acid were within normal limits, and urinalysis was not consistent with a urinary tract infection.

On imaging, ultrasound revealed a complex cystic mass originating from the right upper renal pole (Image A). Evaluation by computed tomography (CT) showed a large subcapsular multicystic mass with hyperdense internal septation on the right kidney suggestive of CE, as well as a small, hypoattenuating lesion in the right lobe of the liver (Image B).^5^ Further imaging by magnetic resonance (MRI) confirmed the subscapular multicystic mass along the right kidney measuring 7 × 5.2 × 6.1 cm consistent with CE stage III and a 1.7 cm cystic lesion in the right hepatic lobe also consistent with CE (Image C). Serum immunoglobulin G (IgG) for echinococcus was positive. The patient was diagnosed with echinococcal disease and was initiated on albendazole 200 milligrams twice a day for 3–4 months, with future plans for surgical intervention.

## DISCUSSION

Echinococcal disease is caused by infection with the *Echinococcus granulosus* tapeworm with the majority of cases originating in the Middle East, South and Central America, and some sub-Saharan African countries. The clinical presentation of *Echinococcus* infection is largely dependent on the location and size of the cysts. Small or calcified cysts may be asymptomatic, whereas larger cysts may cause mass effect, obstruction to blood or urine flow, or may present as toxic-appearing with rupture or secondary bacterial infection.^5^–^7^ Ruptured cysts can cause an anaphylaxis-like reaction of varying severity. Some cysts present with symptoms up to several decades after initial infection or remain asymptomatic indefinitely.^5^–^7^ Our patient had immigrated to the US from Yemen six years earlier, suggesting that her disease has been asymptomatic for a minimum of six years, if not longer.

The most common sites of cystic involvement are the liver (approximately 66%), followed by the lungs (25%). Less commonly reported sites include the brain, kidneys, muscle, bone, and heart.^5^–^7^ Cysts in the kidneys are rare, and have been reported to cause hematuria and flank pain and can potentially result in glomerulonephritis and secondary amyloidosis.^5^–^7^ Our patient’s primary renal cyst was large enough to cause mass effect, leading to dysuria and suprapubic pain. At the time of presentation, she did not complain of hematuria or flank pain.

Generally, diagnosis of echinococcal disease is made with both imaging and serology. With regard to imaging, ultrasonography is 90–95% sensitive for CE, and CT is only moderately better at 95–100% sensitivity; however, CT is superior to ultrasonography for evaluation of extrahepatic cysts. MRI offers no major advantage over CT. Our patient had imaging by all three modalities, with CT and MRI both providing better results on the size and nature of cysts than ultrasonography (Image). When considering serology, antibody detection has greater sensitivity than antigen detection.^9^ Our patient tested positive for echinococcal IgG. IgE and IgM were not pursued as echinococcal IgG is known to have better sensitivity.

Management of these cysts are based on the WHO classification criteria and typically use a combination of observation, albendazole, PAIR (percutaneous puncture, aspiration, injection, re-aspiration), and surgery.^10^ Our patient’s renal cyst was classified as WHO stage III, for which the recommended treatment is albendazole followed by either PAIR or surgery. Alcohol injection was not pursued given the size of the cyst and risk of rupture. She was discharged on albendazole and scheduled follow-up with infectious disease, urology, and general surgery.

**CPC-EM Capsule**

What do we already know about this clinical entity?

Cystic echinococcosis is a parasitic infection that results in the creation of cystic structures in visceral organs, most commonly in the liver or lungs.

What makes this presentation of disease reportable?

We discuss a patient who was diagnosed with cystic echinococcosis of primary renal involvement, a rare anatomic location.

What is the major learning point?

Although these cysts are often found in the liver or lungs, they can arise in almost any organ and symptoms are often specific to the organ system affected.

How might this improve emergency medicine practice?

This case reports a rare presentation of an uncommon disease in the United States and reviews diagnostic and treatment guidelines.

## CONCLUSION

Though echinococcal disease is uncommon in the US, careful attention should be paid in individuals who have emigrated from endemic countries within the prior 10 years given that the mortality of unidentified and untreated individuals is significant. While the liver and lungs are the most common sites of involvement, it is important to note that cysts can be found in almost any anatomic location and the symptoms are often specific to the organ system affected. Cyst rupture can result in an anaphylaxis-like reaction of varying severity. The best imaging modality for extrahepatic cysts in particular is by CT. The majority of these patients should be started on albendazole in the ED, with definitive treatment often requiring evaluation by several subspecialists including those in infectious disease, interventional radiology, and surgery.

## Figures and Tables

**Image:**
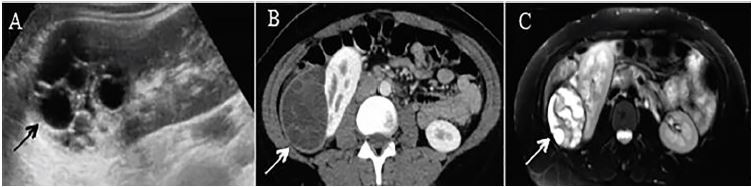
Large, subscapular multicystic renal mass with internal septations as indicated by the arrow on (A) renal ultrasound, (B) computerized tomography, and (C) magnetic resonance imaging T2.
